# Supplementary material for: A global analysis of how human infrastructure squeezes sandy coasts
Source: Nat Commun. 2024 Jan 10;15:432. doi: 10.1038/s41467-023-44659-0 (PMC10781753; doi:10.1038/s41467-023-44659-0)
Supplement: Supplementary file 1 — Supplementary information [file 41467_2023_44659_MOESM1_ESM.pdf]

Supplementary information

A global analysis of how human infrastructure squeezes sandy coasts

Eva M. Lansu[1,2], Valérie C. Reijers[3], Solveig Höfer[1,2], Arjen Luijendijk[4,5], Max Rietkerk[6], Martin J. Wassen[6], Evert Jan Lammerts[7], Tjisse van der Heide[1,2]*

Affiliations

[1] Department of Coastal Systems, Royal Netherlands Institute for Sea Research (NIOZ), Den Burg, The Netherlands.

[2] Conservation Ecology Group, Groningen Institute for Evolutionary Life Sciences, University of Groningen, Groningen, The Netherlands

[3] Faculty of Geosciences, Department of Physical Geography, Utrecht University, Utrecht, The Netherlands

[4] Department of Resilient Ports and Coasts, Deltares, Delft, The Netherlands

[5] Department of Hydraulic Engineering, Faculty of Civil Engineering and Geosciences, Delft University of Technology, Delft, The Netherlands

[6] Copernicus Institute of Sustainable Development, Environmental Sciences Group, Utrecht University; Utrecht, The Netherlands

[7] Programma Deltanatuur, Staatsbosbeheer, Amersfoort, The Netherlands

*Correspondence to: tjisse.van.der.heide@nioz.nl, eva.lansu@nioz.nl

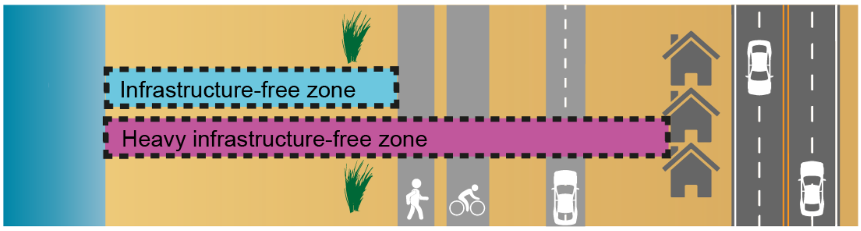

Supplementary Fig. 1. Two proxies to assess the degree of coastal squeeze by human infrastructure. The *infrastructure-free zone* starts at the shoreline and is ended by any paved road or building. The *heavy infrastructure-free zone* also starts at the shoreline, may include paved pedestrian paths, bike lanes or car roads, but is ended by buildings and freeways.

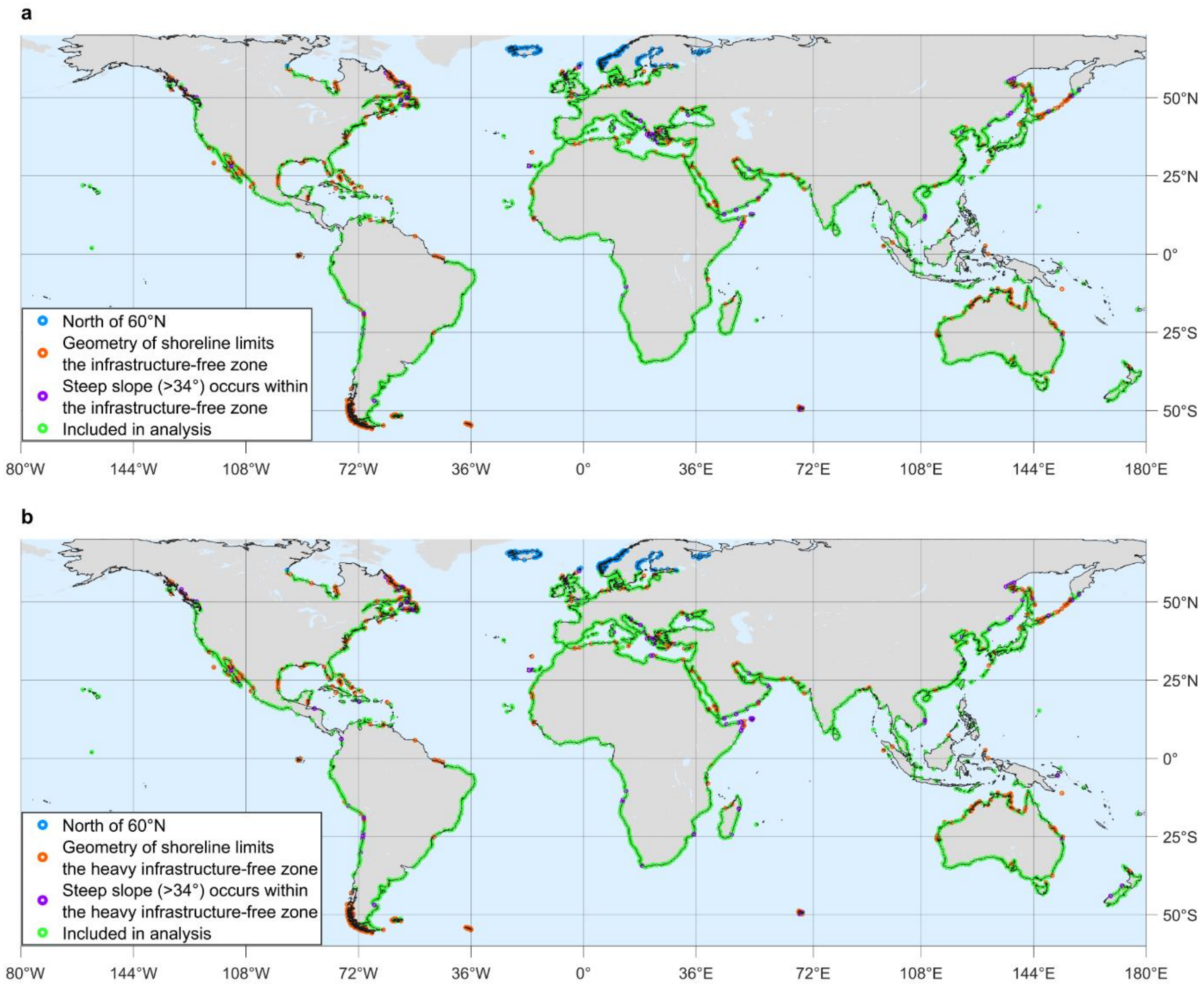

Supplementary Fig. 2. The applied filter criteria on world's sandy shores. Prior to analysis of the infrastructure-free coastal width (**a**) and the heavy infrastructure-free width (**b**) we first omitted transects from latitudes exceeding 60°N, reducing the total number from 253,995 to 235,469 transects. Next, we identified transects where natural limitations occurred prior to intersecting infrastructure due to (1) intersections with the shoreline or (2) slopes steeper than 34°. Exclusion of transects with natural obstructions yielded 168,654 and 150,695 remaining transects for further analysis of the infrastructure-free and heavy infrastructure-free coastal width, respectively. Matlab mapping toolbox was used to create the figure. Source data are provided as a Source Data file.

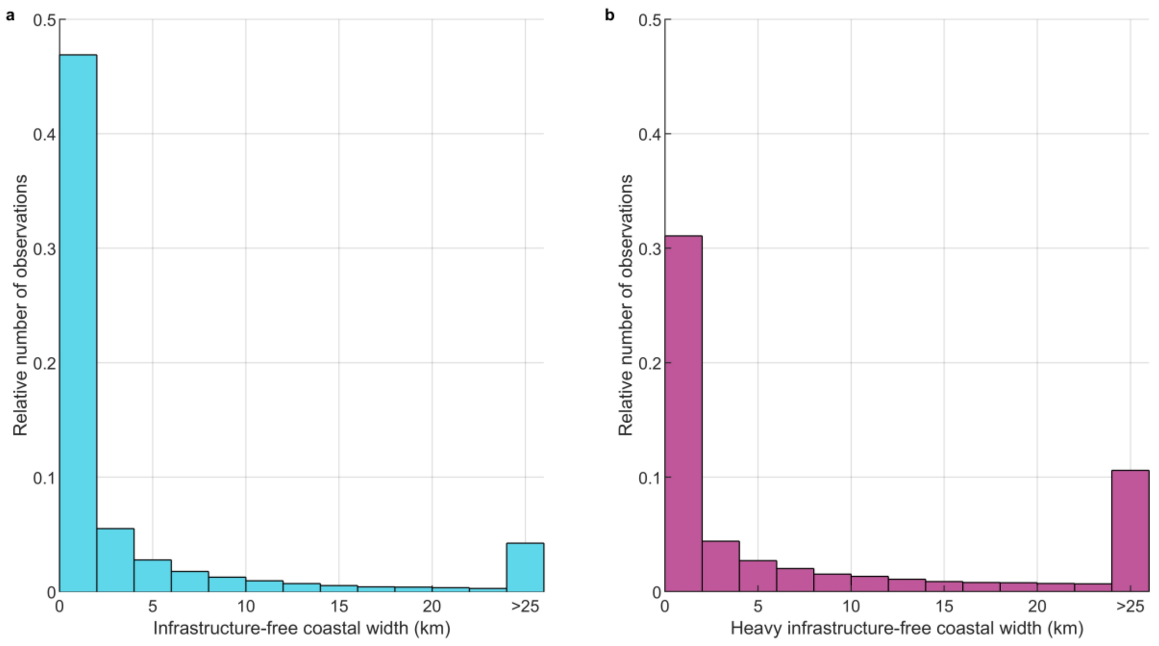

Supplementary Fig. 3. Frequency distributions of the unimpacted coastal width. The frequency distribution of the *infrastructure-free zone* (**a**) and the *heavy infrastructure-free zone* (**b**) are both positively skewed with the nearest structure most often found close to shore. Source data are provided as a Source Data file.

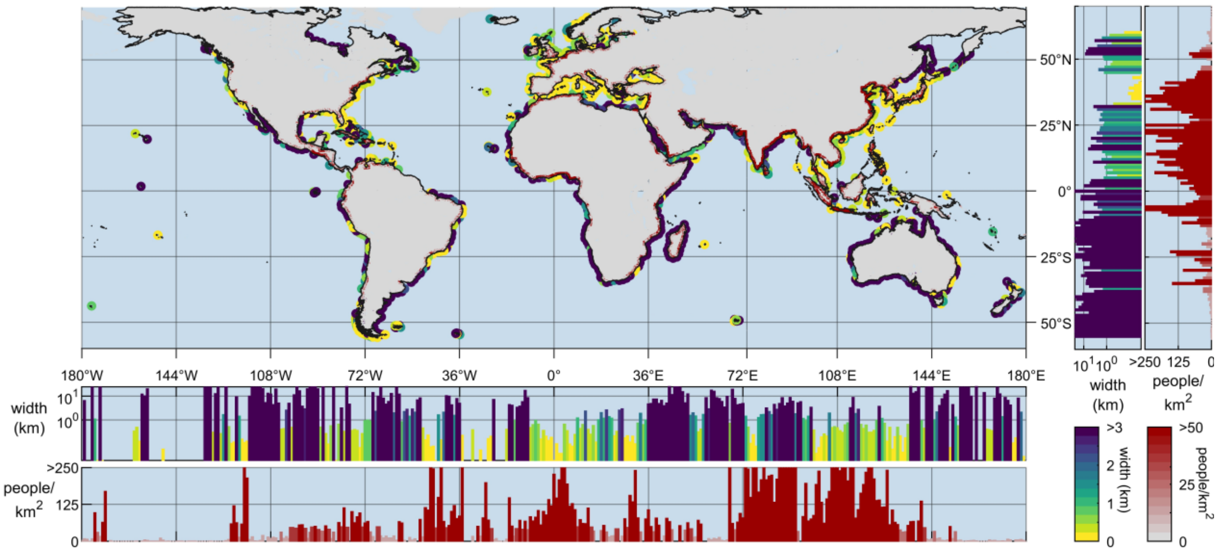

Supplementary Fig. 4 Map of the coastal squeeze by heavy infrastructure along the world's sandy shores. Heavy infrastructure-free width is depicted in yellow-green-blue from 0 to 3 km. Coastal population density (data obtained from WorldPop[64]) is depicted in white-red from 0 to 50 people/km^2. Bar graphs show the latitudinal and longitudinal averages. Human infrastructure is generally closer to sandy shores in more densely populated areas, particularly between 32 and 45 degrees North. Matlab mapping toolbox was used to create the figure. Source data are provided as a Source Data file.

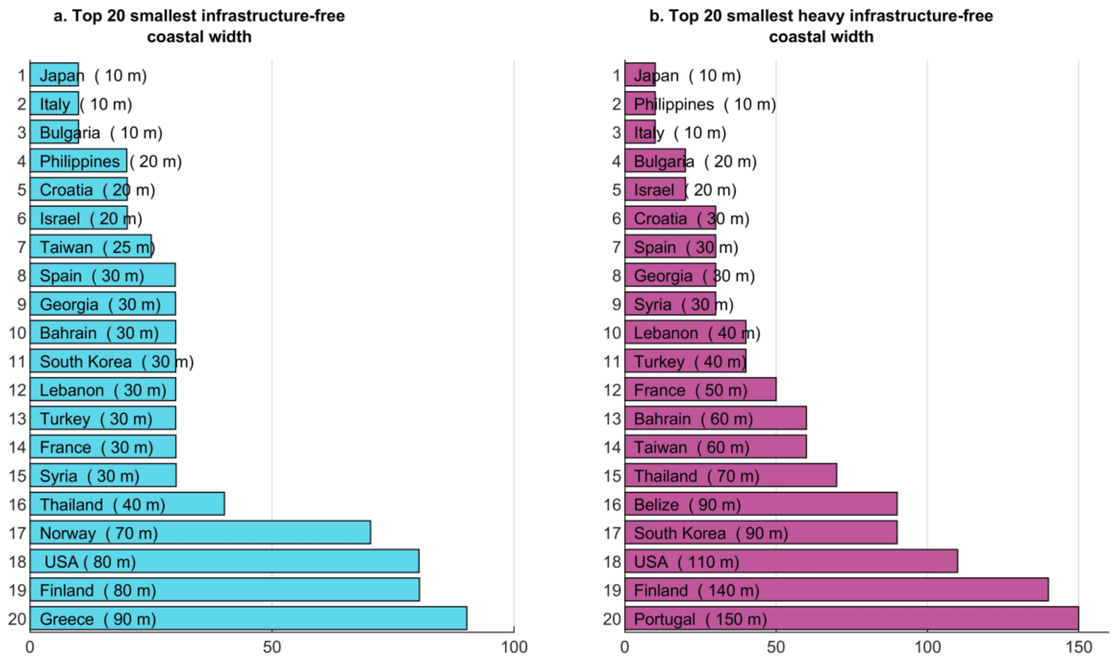

Supplementary Fig. 5. Ranking of the top 20 countries with the most squeezed sandy coasts. The median coastal width is presented as bar and as value between brackets. Panel **a** shows the infrastructure-free coastal width, panel **b** the heavy infrastructure-free coastal width. Note that, to focus on countries with a substantial portion of sandy coastline, we excluded countries with less than 100 km of sandy coast in this overview. Source data are provided as a Source Data file.

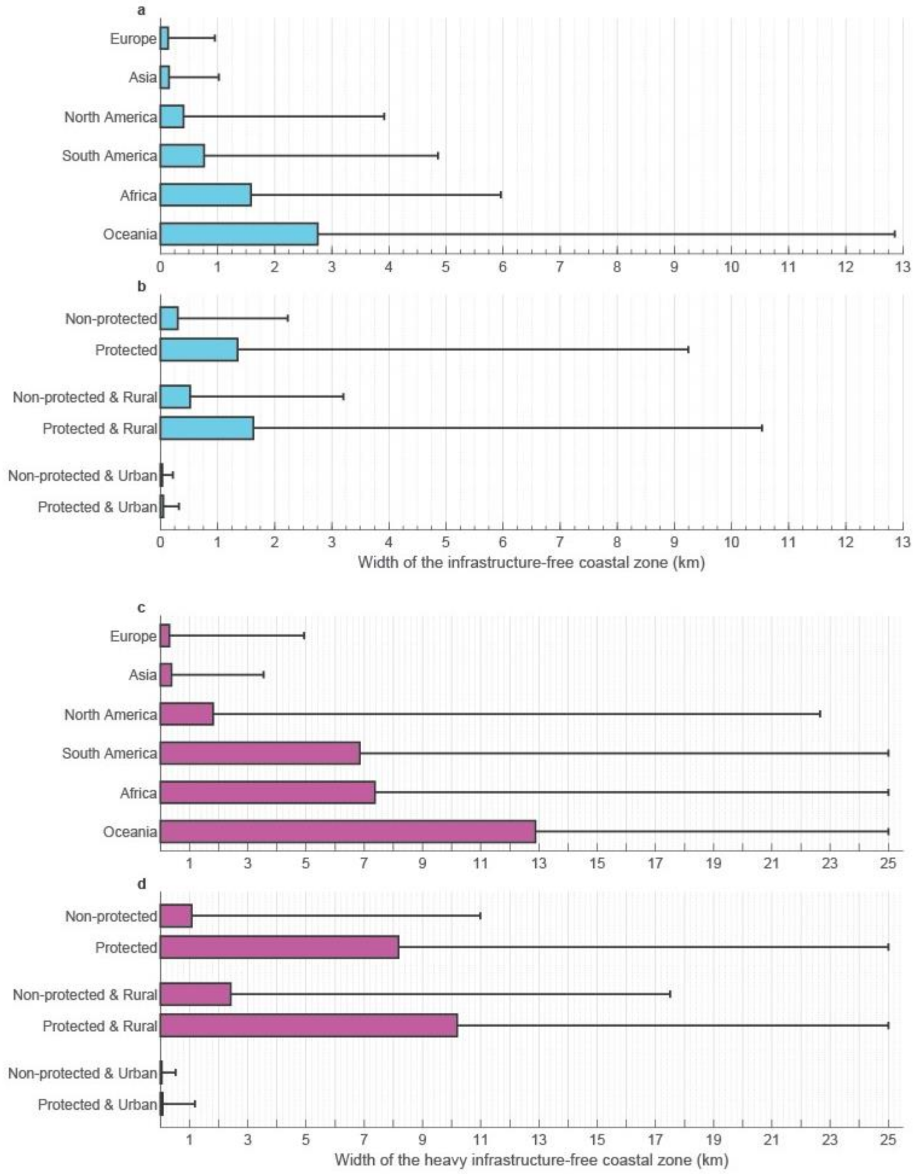

Supplementary Fig. 6. Infrastructure-mediated coastal width by continent and inside/outside nature reserves. The width of the infrastructure-free (**a**) and heavy infrastructure-free (**c**) sandy shore per continent. The width of the infrastructure-free (**b**) and heavy infrastructure-free (**d**) sandy shore in nature protected versus non-protected areas. For all data combined, the infrastructure-free width (Wilcoxon rank-sum test: Z= 71.6, p<0.001) and the heavy infrastructure-free width (Wilcoxon rank-sum: Z= 71.2, p<0.001) are significantly larger in nature reserves. Moreover, when distinguishing between urban (non) protected and rural (non) protected areas, groups were found to have different distributions (Kruskal-Wallis test for infrastructure-free: χ^2=22189, p<0.001, df=3; heavy infrastructure-free: χ^2= 23712, p<0.001, df=3). Additional posthoc analysis demonstrated that all groups differed from each other. Bars represent medians and whiskers 75th percentiles. Source data are provided as a Source Data file.

Supplementary Table 1. Results of multiple linear regression models to explain the country medians of the infrastructure-free coastal width (**a**) and the heavy infrastructure-free width (**b**) with coastal population density and GDP per capita. Source data are provided as a Source Data file.

a. Multiple linear regression: infrastructure-free width ~ population density + GDP

Coefficients	St. Estimate	SE	tStat	pValue
Intercept	-0.06	0.05	-1.12	0.27
Population density	-0.39	0.05	-7.38	1.02e-11
GDP	-0.33	0.05	-6.48	1.29e-09
Number of Observations	152			
R^2	0.35			
$R^2_{adjusted}$	0.34			

b. Multiple linear regression: heavy infrastructure-free width ~ population density + GDP

Coefficients	St. Estimate	SE	tStat	pValue
Intercept	-0.01	0.06	-0.24	0.81
Population density	-0.50	0.06	-7.73	1.54e-12
GDP	-0.45	0.06	-7.37	1.12e-11
Number of Observations	151			
R^2	0.39			
$R^2_{adjusted}$	0.38			

Supplementary Table 2 The percentage of sandy shores that would completely lose their infrastructure-free space to projected coastal retreat by 2100. We subtracted the projected retreat from the infrastructure-free coastal width (**a**) and heavy infrastructure-free coastal width (**b**) under both Representative Concentration Pathway 4.5 and 8.5. Source data are provided as a Source Data file.

a. Infrastructure-free width exceeded by coastal retreat (%)

	RCP 4.5	RCP 8.5
Africa	11.4	13.5
Asia	28.0	33.2
Europe	30.7	36.3
North America	21.6	26.4
Oceania	12.3	15.4
South America	17.8	21.4
Global; width < 0 m	22.5	30.1
Global; width < 100 m	34.1	41.2

b. Heavy infrastructure-free width exceeded by coastal retreat (%)

	RCP 4.5	RCP 8.5
Africa	8.2	9.6
Asia	23.3	27.3
Europe	27.4	31.7
North America	17.4	21.0
Oceania	10.0	12.0
South America	12.0	14.1
Global; width < 0 m	18.5	21.7
Global; width < 100 m	27.0	28.8

Supplementary Table 3. Overview of data sources used in this study. Dataset projections were transformed to WGS84 prior to analyses.

Source	Dataset	Resolution	Year	Link
OpenStreetMap	Streets	vector	2020	https://www.openstreetmap.org/#map=7/52.154/5.295
OpenStreetMap	Shoreline	vector	2023	https://osmdata.openstreetmap.de/data/land-polygons.html
Earth Observation Center	Global Urban Footprint	12 m	2015	https://doi.org/10.1016/j.isprsjprs.2017.10.012
Deltares & TU Delft	Identification of the sandy coastline	500 m alongshore	2016	10.1038/s41598-018-24630-6
Worldpop	The spatial distribution of population in 2020	1 km	2020	https://hub.worldpop.org/doi/10.5258/SOTON/WP00647
World Bank Group	GDP per capita	One value per country	2019	https://data.worldbank.org/indicator/NY.GDP.PCAP.CD
IUCN and UNEP-WCMC	Terrestrial Protected Areas	vector	2021	https://www.protectedplanet.net/en/thematic-areas/wdpa?tab=WDPA
European Commission, Joint Research Centre (JRC)	Global shoreline change projections	500 m alongshore	2020	https://data.jrc.ec.europa.eu/dataset/18eb5f19-b916-454f-b2f5-88881931587e
Climate Central	CoastalDEM	90 m	2021	https://www.climatecentral.org/coastaldem-v2.1
